# Characterization of copper-resistant bacteria and bacterial communities from copper-polluted agricultural soils of central Chile

**DOI:** 10.1186/1471-2180-12-193

**Published:** 2012-09-05

**Authors:** Fabiola Altimira, Carolina Yáñez, Guillermo Bravo, Myriam González, Luis A Rojas, Michael Seeger

**Affiliations:** 1 Laboratorio de Microbiología Molecular y Biotecnología Ambiental, Departamento de Química and Center of Nanotechnology and Systems Biology, Universidad Técnica Federico Santa María, Avenida España 1680, Valparaíso, Chile; 2 Instituto de Biología, Facultad de Ciencias, Pontificia Universidad Católica de Valparaíso 1680, Valparaíso, Chile

**Keywords:** Copper-resistant bacteria, *copA* gene, *Sphingomonas*, *Stenotrophomonas*, *Arthrobacter*, Bacterial communities

## Abstract

**Background:**

Copper mining has led to Cu pollution in agricultural soils. In this report, the effects of Cu pollution on bacterial communities of agricultural soils from Valparaiso region, central Chile, were studied. Denaturing gradient gel electrophoresis (DGGE) of the 16S rRNA genes was used for the characterization of bacterial communities from Cu-polluted and non-polluted soils. Cu-resistant bacterial strains were isolated from Cu-polluted soils and characterized.

**Results:**

DGGE showed a similar high number of bands and banding pattern of the bacterial communities from Cu-polluted and non-polluted soils. The presence of *copA* genes encoding the multi-copper oxidase that confers Cu-resistance in bacteria was detected by PCR in metagenomic DNA from the three Cu-polluted soils, but not in the non-polluted soil. The number of Cu-tolerant heterotrophic cultivable bacteria was significantly higher in Cu-polluted soils than in the non-polluted soil. Ninety two Cu-resistant bacterial strains were isolated from three Cu-polluted agricultural soils. Five isolated strains showed high resistance to copper (MIC ranged from 3.1 to 4.7 mM) and also resistance to other heavy metals. 16S rRNA gene sequence analyses indicate that these isolates belong to the genera *Sphingomonas*, *Stenotrophomonas* and *Arthrobacter*. The *Sphingomonas* sp. strains O12, A32 and A55 and *Stenotrophomonas* sp. C21 possess plasmids containing the Cu-resistance *copA* genes. *Arthrobacter* sp. O4 possesses the *copA* gene, but plasmids were not detected in this strain. The amino acid sequences of CopA from *Sphingomonas* isolates (O12, A32 and A55), *Stenotrophomonas* strain (C21) and *Arthrobacter* strain (O4) are closely related to CopA from *Sphingomonas*, *Stenotrophomonas* and *Arthrobacter* strains, respectively.

**Conclusions:**

This study suggests that bacterial communities of agricultural soils from central Chile exposed to long-term Cu-pollution have been adapted by acquiring Cu genetic determinants. Five bacterial isolates showed high copper resistance and additional resistance to other heavy metals. Detection of *copA* gene in plasmids of four Cu-resistant isolates indicates that mobile genetic elements are involved in the spreading of Cu genetic determinants in polluted environments.

## Background

Copper is widely distributed in nature and it is often found in the Earth’s crust. Cu is an essential trace element for living organism, playing a role in an important number of biological processes [1,2]. The properties of the metallic form of copper, such as its electricity and heat conductivity, resistance to corrosion, malleability and ductility, have been useful for a wide variety of applications. Elevated levels of Cu from natural and industrial sources have been reported in several Cu-producing countries such as Chile, China, Indonesia, Russia, Zambia, and Australia [3-8]. The mining activities and the use of pesticides to control plant diseases have increased the Cu levels in agricultural soils. Cu could bind to soil components (organic matter, clay minerals, Fe, Al and Mn oxides) leading a significant accumulation in the soil surface [9]. Soil bacteria are responsible for diverse ecological processes, such as biochemical cycling of the elements, plant growth, decomposition of organic matter, maintenance of soil structure, detoxification and pest control [10-13]. Cu accumulation could induce harmful effects on soil bacteria damaging the biological processes and the soil quality [10,14,15].

Culture independent molecular techniques such as DGGE have been used to study microbial communities. DGGE is based on an amplification step with specific primers to amplify a specific gene (for example, a highly variable region of the 16S rRNA gene) and a denaturing gradient polyacrylamide gel electrophoresis. Studies of soil bacterial community by DGGE revealed that heavy metal contamination in agricultural soils close to copper and zinc smelters may provoke changes in the composition of soil bacterial community and a decrease of the bacterial diversity [11,16]. However, changes in the soil bacterial community exposed to heavy metal may vary depending of soil properties, heavy metal bioavailability and the indigenous microbial groups in soil [9].

The genes conferring copper resistance in bacteria are often present in plasmids and organized in an operon [17-19]. The copper resistance is encoded by the *cop* genes (*copA*, *copB*, *copC* and *copD*) in *Cupriavidus metallidurans* CH34, *Pseudomonas syringae* pv. *tomato* PT23*, Xanthomonas axonopodis* pv. *vesicatoria* E3C5 and *Pseudomonas aeruginosa* PAO1 and by the *pco* genes (*pcoA*, *pcoB*, *pcoC* and *pcoD*) in *Escherichia coli* strain RJ92 [20-24]. The *copA* gene encoding a multi-copper oxidase (*pcoA* gene in *E. coli*) is one of the main genetic determinants involved in Cu-resistance in Gram-negative bacteria. It encodes the multi-copper oxidase that oxidase Cu(I) to the less toxic chemical form of Cu(II) [1,25,26]. A different *copA* gene that encodes a Cu-transporting P-type ATPase involved in Cu homeostasis has been described in *E. coli* and other bacteria [17]. The *copA* gene encoding a multi-copper oxidase is widely present in Cu-resistant bacterial strains and may represent a relevant marker to study the Cu-resistance in bacteria [26]. The aims of this study were to investigate the effect of long-term Cu pollution on the bacterial community and the characterization of Cu-resistant bacteria from agricultural sites located close to copper smelters from the Aconcagua valley, central Chile.

## Methods

### Chemicals

The metal salts CuSO_4_·5H_2_O, ZnCl_2_, K_2_CrO_4_, NiCl_2_·H_2_O, HgCl_2_, CoCl_2_·6H_2_O, CdCl_2_·2H_2_O (analytical grade) were purchased from Merck (Darmstadt, Germany) and used to prepare stock solutions of Cu^2+^, Zn^2+^, CrO_4_^2-^, Ni^2+^, Co^2+^, Cd^2+^ (800 mM), and Hg^2+^ (150 mM). HNO_3_, HClO_4_ and H_2_SO_4_ (Suprapur) and standard Titrisol solution were obtained from Merck (Darmstadt, Germany). *Taq* DNA polymerase and bovine serum albumin for PCR were obtained from Invitrogen (Carlsbad, CA, USA). *Taq* DNA polymerase Stoffel fragment was obtained from Applied Biosystems (Darmstadt, Germany). Tryptic soy broth (TSB) and R2A medium were purchased from BD Diagnostic Systems (Heidelberg, Germany). Formamide and ammonium persulfate (APS), *N*,*N*,*N*′,*N*′-tetramethylethylenediamine (TEMED) were purchased from Sigma-Aldrich (St. Louis, MO, USA) and urea from Bio Rad (Hercules, CA, USA). Acrylamide was obtained from Winkler (Santiago, Chile).

### Soil sampling

**Figure 1:**
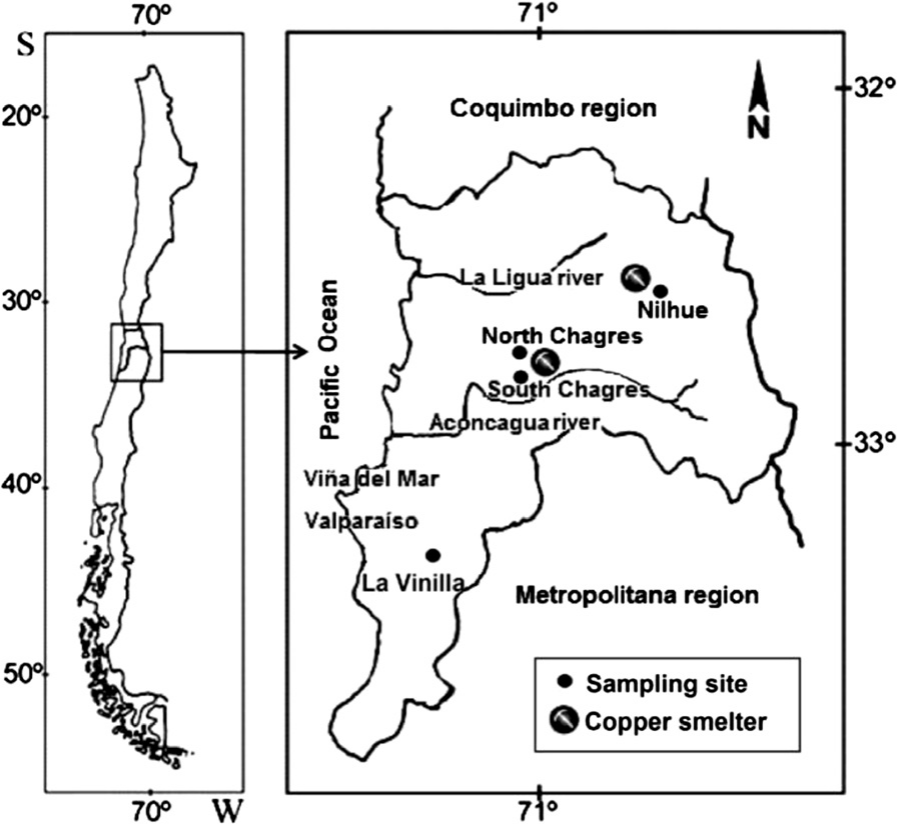
**Location of sampling sites of agricultural soils in Valparaíso region, central Chile.** North Chagres, South Chagres and Ñilhue are Cu-polluted sites. La Vinilla is a non-polluted site.

Three composite soil samples were collected from four different agricultural sites in Valparaiso region (central Chile). Each composite sample contained 12 bulk soil cores from the surface stratum (0–10 cm depth) taken from three sampling points located in an area of 250 m^2^ per site. The three Cu-polluted sites were located in the neighborhood of an active or an abandoned Cu smelter in the Aconcagua valley (Figure 1). These soils were sampled from a pasture soil located in North Chagres (longitude 70º57’29.95” W and latitude 32º46’37.42” S), an artichoke plantation soil from South Chagres (longitude 70º57’57.169” W and latitude 32º48’30.254” S) located 3.5 km distant from North Chagres site and an olive plantation soil from Ñilhue (longitude 70º54’40.628” W and latitude 32º41’44.577” S) located 10.8 and 13.5 km distant from North and South Chagres sites, respectively. Soils were sampled on 6 August 2009. These soils had a Cu content that ranged from 379 to 784 mg kg^-1^ dry weight soil (*d.w.s*). The concentrations exceed the standard acceptable level of 40 mg kg^-1^ for soil by the Québec regulatory authorities (Ministère de l’ Environnement du Québec, 1999). A pasture soil from a non-polluted site was sampled from the Casablanca valley, central Chile on 5 August 2010. The non-polluted site was located in La Vinilla (longitude 71º24’36” W and latitude 32º19’30.254” S) located 62–68 km distant from the three polluted sites. Soil samples were air-dried and sieved to 2 mm and homogenized. The soil samples were stored in polyethylene bags and preserved in a dark room at 4°C until analyses.

### Soil chemical analyses

Soil pH was measured using a 1:2 (w/v) a soil/deionized water mixture. The organic matter content was determined by the dichromate oxidation [27]. For heavy metal analyses (Cu, Zn, Pb, Cr and Ni), soils were digested with a 10:4:1 HNO_3_/HClO_4_/H_2_SO_4_ mixture. Exchangeable Cu from soils (1 g *d.w.s*) was extracted with 10 ml of MgCl_2_ solution (1 M, pH 7) at room temperature with continuous agitation for 1 h. Total heavy metal content and the exchangeable Cu were quantified by atomic absorption spectrometry (AAS) using Spectraa-800 spectrophotometer Varian (Santa Clara, CA, USA).

### DNA extraction from soil

Metagenomic DNA was extracted from 0.5 g of soil in triplicate using the FastDNA Spin Kit for soil (MP Biomedicals, Solon, Ohio, USA). Cells were disrupted using the FastPrep-24 instrument (MP Biomedicals, Solon, Ohio, USA) following the manufacturer's instructions. Subsequently, the DNA extract was purified by GeneClean Spin Kit (MP Biomedicals, Solon, Ohio, USA). DNA was quantified using Qubit fluorometer (Invitrogen, Carlsbad, CA, USA).

### Bacterial community analyses

Bacterial communities from soils were evaluated using DGGE. The universal primers 27f (5′-AGAGTTTGATCMTGGCTCAG-3′) and 1492r (5′-TACGGYTACCTTGTTACGACTT-3′) were used for PCR amplification of 16S rRNA genes followed by a nested DGGE-PCR targeting the V6 region of the 16S rRNA genes using GC-F968-984 (5′-CGCCCGGGGCGCGCCCCGGGCGGGGCGGGGCACGGGGGGAACGCAAGACCTTAC-3′) and R1378-1401 (5′-CGGTGTGTACAAGGCCCGGGAACG-3′) primers [28]. Amplification of 16S rRNA gene was conducted in a volume of 25 μl containing F27 and R1492 primers (0.6 μM), deoxyribonucleoside triphosphate (400 μM each), PCR buffer, *Taq* DNA polymerase (2.5 U), MgCl_2_ (3.0 mM), bovine serum albumin (0.1 mg ml^-1^), soil DNA template (20 ng) and ultra pure water. DNA amplification was performed in an Eppendorf Mastercycler thermocycler (Hamburg, Germany) using the following conditions: 1 cycle of 94°C for 5 min, and 25 cycles of 94°C for 1 min, 55°C for 1 min, 72°C for 2 min, plus a final extension at 72°C for 10 min. The amplification of V6 region was conducted using, GC-F968-984 and R1378-1401 primers (0.6 μM), deoxyribonucleoside triphosphate (200 μM each), Stoffel buffer, *Taq* DNA polymerase Stoffel fragment (2.5 U), MgCl_2_ (3.0 mM), and bovine serum albumin (0.4 mg ml^-1^), 1 μl template DNA (obtained from a 1:10 dilution of 16S rRNA amplicon) and ultra pure water. DNA amplification was carried out using the following conditions: 1 cycle of 94°C for 5 min, and 20 cycles of 94°C for 1 min, 55°C for 1 min, 72°C for 1 min, plus a final extension at 72°C for 10 min.

DGGE was performed using the method previously reported [28] with minor modifications. The BioRad DCode DGGE system was used with an 8% (w/v) polyacrylamide gel containing a denaturing gradient from 30% to 60% (100% denaturant contains 40% (v/v) formamide and 7 M urea). Equal amounts of DNA were loaded on each well. Amplicons were separated at constant voltage of 70 V for 13 h at 58°C. The gel was stained with GelRed (Biotium Inc., Hayward, CA, USA) 1:10,000 (v/v) for 30 min, digitally photographed under UV light and analyzed in a Gel Doc XR System (Bio-Rad, Hercules, CA, USA).

Bands of DGGE profiles were analyzed by using the software Phoretix 1D v11.2 (Non Linear Dynamics, Newcastle, UK). Background noise was subtracted by rolling ball algorithm with a radius of 50 pixels; the automatic band detection was performed with a minimum slope of 100 and a noise reduction of 5, and peaks smaller than 2% of the maximum peak were discarded. Bands were manually corrected and matched to create an absent/present binary matrix. A dendrogram was constructed by Unweighted Pair Group Method with Arithmetic Mean (UPGMA), clustering using percentage of similarity averages with MultiVariate Statistical Package (MVSP) version 3.13 h (GeoMem, Blairgowrie, United Kingdom). The diversity of bacterial communities were determined by the Shannon index (*H’*) that considers the total number of species in a bacterial community (*S*, richness) and the frequency of the species (abundance). The richness of bacterial community was determined by the number of bands present in DGGE profiles of soils [15]. Three soil replicates were analyzed for each DGGE soil sample.

### Detection of *copA* gene in metagenomic DNA from soils

Metagenomic DNA extracted from each soil was used for *copA* gene amplification. The degenerated primers forward Coprun F2 (5’-GGSASDTACTGGTRBCAC-3’) and reverse Coprun R1 (5’-TGNGHCATCATSGTRTCRTT-3’) [26] were used for *copA* gene amplification from soil metagenomic DNA. Degenerated Coprun primers were designed for the amplification of *copA* genes that encode the multi-copper oxidase from *Proteobacteria*. DNA amplification was performed using the following conditions: 1 cycle of 94°C for 3 min, 35 cycles of 94°C for 1 min, 58°C for 1 min, 72°C for 1 min, plus a final extension at 72°C for 7 min.

### Enumeration of heterotrophic bacteria and isolation of Cu-tolerant bacteria from soils

Bacterial cells were extracted from 1 g of each soil suspended in 9 ml of phosphate buffer (50 mM, pH 7) and vigorously shaken in an orbital shaker (200 rpm) for 30 min. After decantation for 1 min, serial dilutions were prepared from the supernatant. The total cultivable heterotrophic bacteria were grown in R2A medium supplemented with cycloheximide (100 mg l^-1^) [29]. The Cu-tolerant bacteria were grown in same conditions supplemented with Cu^2+^ (0.8 mM) [30]. Ninety two bacterial strains (29 to 31 from each polluted soil) were isolated based on their capability to grow in presence of Cu^2+^ (0.8 mM) and the colony morphology. Statistical analysis was performed using one-way ANOVA (OriginPro 8 for Windows). Differences were considered to be significant at *P* ≤ 0.05.

### Minimum inhibitory concentration (MIC) of Cu and other heavy metals for bacterial strains

Bacterial isolates were grown in diluted (1:10) TSB liquid medium. An aliquot (10 μl) of each culture grown until stationary phase were placed onto the agar plates with low phosphate Tris mineral salts (LPTMS) medium [31], supplemented with Cu^2+^ concentrations ranged from 0.8 to 4.7 mM (in increasing concentration of 0.4 mM steps). Inoculated plates were incubated at 30°C and checked for growth after 72 h. Experiments were done in duplicate. The lowest heavy metal concentration that prevented growth was recorded as the MIC [31].

The MIC values to Co^2+^, Ni^2+^, Zn^2+^, Cd^2+^, Hg^2+^ and CrO_4_^2-^ were studied in bacterial isolates that were capable to grow in presence of Cu^2+^ (2.8 mM). LPTMS medium supplemented with different concentrations of each heavy metal was used following a protocol previously described [31]. The concentrations of Co^2+^ ranged from 0.8 to 6.8 mM (in increasing concentration of 0.2 mM steps), Ni^2+^ ranged from 0.8 to 17 mM (in increasing concentration of 0.3 mM steps), Zn^2+^ ranged from 0.8 to 17 mM (in increasing concentration of 0.3 mM steps), Cd^2+^ ranged from 0.4 to 3.6 mM (in increasing concentration of 0.2 mM steps), Hg^2+^ ranged from 0.005 to 0.5 mM (in increasing concentration of 0.025 mM steps), and CrO_4_^2-^ ranged from 0.4 to 8.6 mM (in increasing concentration of 0.2 mM steps). The plates were incubated at 30°C for 72 h. The MIC analyses were done in duplicate.

### PCR amplification of 16S rRNA and heavy metal resistance genes from bacterial isolates

PCR reactions were conducted in a volume of 25 μl containing specific primers (0.6 μM each), deoxyribonucleoside triphosphate (400 μM each), PCR buffer, *Taq* polymerase (1.25 U), MgCl_2_ (3 mM). Genomic DNA was prepared from single colonies re-suspended in 100 μl of Tris-EDTA buffer (TE, pH 7.5), heated at 95°C for 5 min and centrifuged briefly. The supernatant (2 μl) was used for PCR reactions. The universal primers forward 27f and reverse 1492r were used for 16S rRNA gene amplification. The primers forward Coprun F2 and reverse Coprun R1 were used for the amplification of the *copA* gene. The forward primer 5’-GTCGTTAGCTTGCCAACATC-3’ and the reverse primer 5’-CGGAAAGCAAGATGTCGAATCG-3’ [31] were used for *chrB* gene (chromate resistance) amplification. The forward primer 5’-ACCATCGGCGGCACCTGCGT-3’ and the reverse primer 5’-ACCATCGTCAGGTAGGGGAACAA-3’ were used for *merA* gene (inorganic mercury resistance) amplification [32]. The forward primers 5’-TCGCCCATATATTTTAGAAC-3’ and the reverse primer 5’-GTCGGGACAGATGCAAAGAAA-3’ were used for *merB* gene (organic mercury resistance) amplification [32]. DNA amplification of *chrB*, *merA* and *merB* was carried out using the following conditions: 1 cycle of 94°C for 3 min, 30 cycles of 94°C for 1 min, 57°C for 1 min, 72°C for 1 min, plus a final extension at 72°C for 7 min. *C. metallidurans* MSR33 was used as positive control for *copA*, *chrB*, *merA* and *merB* genes [31]. PCR products were visualized by agarose gel electrophoresis followed by staining with GelRed (1:10,000 v/v).

### 16S rRNA and *copA* genes sequence analyses

The PCR products were visualized by agarose gel electrophoresis. Bands were cut from the gel with a scalpel and DNA was recovery using Zimoclean Gel DNA Recovery Kit (Irvine, CA, USA). The purified DNA was sequenced directly by an Applied Biosystem 3730XL DNA sequence (Carlsbad, CA, USA), using the primers 27f and 1492r for 16S rRNA gene and Coprun F2 and Coprun R1 for *copA* gene sequencing, respectively. The nucleotide sequences of 16S rRNA genes were aligned with sequences available in the GenBank (http://www.ncbi.nlm.nih.gov/). The nucleotide sequence of *copA* gene was translated into a protein sequence using blastx. Then, partial sequences of CopA were aligned with other CopA sequences from Cu-resistant bacteria [18]. A phylogenetic analysis was performed to study the evolutionary relationships of the sequences based on the alignments calculated by CLUSTAL W using the default options. The evolutionary history was inferred using the Neighbor-Joining method. Evolutionary analyses were conducted in MEGA 5.05 software [33]. The 16S rRNA gene sequence of strains O12, A32, A55, C21 and O4 were submitted to the EMBL Nucleotide Sequence Database under accession number EMBL:HE608567, EMBL:HE608568, EMBL:HE608569, EMBL:HE608570 and EMBL:HE608571, respectively. The *copA* gene sequence of strains O12, A32, A55, C21 and O4 were submitted to the EMBL Nucleotide Sequence Database under accession number EMBL:HE716432, EMBL:HE716433, EMBL:HE16434, EMBL:HE16435, EMBL:HE16436, respectively.

### Plasmid isolation from bacterial strains

Plasmid isolation from bacterial strains was performed using the method of Kado and Liu [34] with minor modifications [31]. Plasmids were visualized in agarose gel electrophoresis. Bands were cut from the gel with a scalpel and plasmids were recovery from the gel using Zimoclean Gel DNA Recovery Kit (Irvine, CA, USA). The presence of *copA* gene in plasmids was assessed by PCR using the protocol described above.

## Results

The bacterial communities of three Cu-polluted agricultural soils and one non-polluted soil from Valparaíso region, central Chile, were characterized. The three polluted agricultural sites from Aconcagua valley are located close to an active or an abandoned Cu smelter. An agricultural soil located far away from mining activities in Casablanca valley was selected as a non-polluted site. Soils from Aconcagua valley (loam) and from Casablanca valley (sandy loam) were neutral. Soils from South Chagres and Ñilhue showed higher organic matter content (4.5%) than soils from North Chagres and La Vinilla (2.3%). The total Cu concentrations of the Aconcagua valley soils ranged from 379 to 784 mg kg^-1^, whereas the total Cu concentration in the La Vinilla soil was only 21 mg kg^-1^. The exchangeable Cu concentration of the North and South Chagres soils was 2.0 and 1.9 mg kg^-1^, respectively, and 1.2 mg kg^-1^ for the Ñilhue soil. The exchangeable Cu concentration observed in the La Vinilla soil was below the detection limit (0.1 mg kg^-1^). The total concentrations of Zn (ranged from 97 to 205 mg kg^-1^), Pb (ranged from 33 to 73 mg kg^-1^) and Cr (ranged from 13 to 19 mg kg^-1^) in Cu-polluted soils from Aconcagua valley were high, whereas in La Vinilla soil heavy metals were present at low concentration.

### Bacterial community profiling in agricultural soils by DGGE

**Figure 2:**
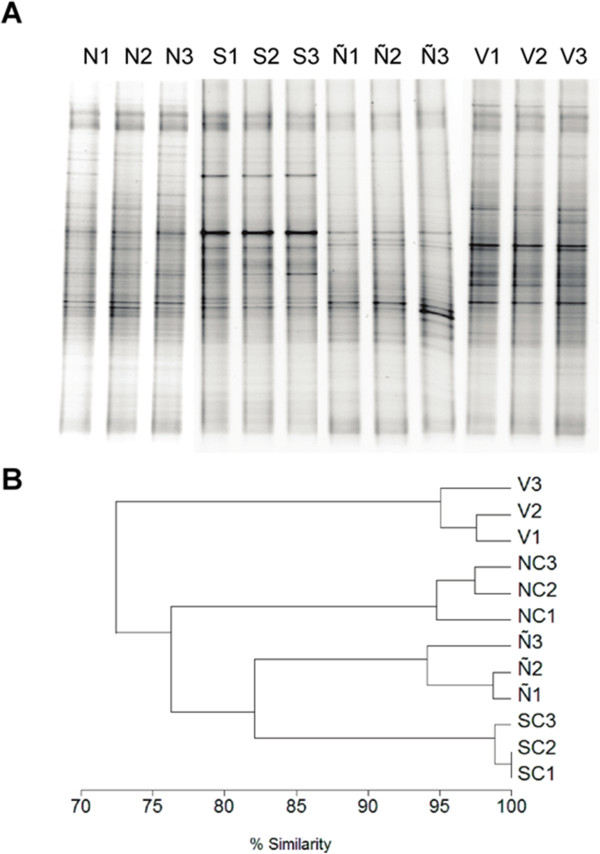
**DGGE of 16S rRNA genes of bacterial communities from agricultural soils.A**. DGGE of bacterial communities from North Chagres (lanes N1-N3), South Chagres (lanes S1-S3), Ñilhue (lanes Ñ1-Ñ3) and La Vinilla (V1-V3). **B**. Dendrogram showing cluster analysis of 16S rRNA gene profiles of bacterial communities from the four agricultural soils. Gel image analysis was performed by using Phoretix 1D software package. Bands were automatically detected and manually corrected. A binary matrix was generated by presence or absence bands. The sample similarities were analyzed by MVSP.

DGGE from the four soils showed complex profiles suggesting a high diversity of the bacterial community in Cu-polluted and non-polluted soils (Figure 2A). UPGMA analysis of banding patterns from bacterial DGGE profiles of the four agricultural sites were grouped into four clusters (Figure 2B). Replicates from each agricultural soil showed a very high similarity (approximately 95%). Soils from South Chagres, Ñilhue and North Chagres showed a high similarity (approximately 80%). The non-polluted La Vinilla soil showed a similarity of 73% with the Cu-polluted soils (Figure 2B). The values of Shannon index obtained for each soil were 3.65 ± 0.01 for North Chagres, 3.77 ± 0.01 for South Chagres, 3.65 ± 0.01 for Ñilhue and 3.71 ± 0.03 for La Vinilla. The richness values (*S*) obtained for each soil were 38.67 ± 0.58 for North Chagres, 43.67 ± 0.58 for South Chagres, 38.33 ± 0.58 for Ñilhue and 40.67 ± 1.15 for La Vinilla (Figure 2B).

### PCR detection of Cu-resistance genes in metagenomic DNA from agricultural soils

The presence of the *copA* gene in the metagenomic DNA from the four agricultural soils was studied. The *copA* gene was detected by PCR in the three Cu-polluted soils from Aconcagua valley (data not shown). In contrast, the *copA* gene was not detected in the non-polluted soil from Casablanca valley.

### Copper tolerance of bacterial community

The Cu-tolerance of the bacterial community of the agricultural soils was determined. The cultivable heterotrophic bacteria ranged from 1.2 × 10^7^ to 2.2 × 10^7^ CFU g^-1^*d.w.s* in Cu-polluted and non-polluted soils. The Cu-tolerant culti-vable bacteria ranged from 3 to 23% (from 7.4 × 10^5^ to 2.8 × 10^6^ CFU g^-1^*d.w.s*) of the total cultivable heterotrophic bacteria in Cu-polluted agricultural soils from Aconcagua valley. In the non-polluted soil from La Vinilla, the Cu-tolerant bacteria were 0.4% (5.9 × 10^4^ CFU g^-1^*d.w.s*). The number of Cu-tolerant cultivable bacteria was significantly larger in Cu-polluted soils than in non-polluted soil (*P* ≤ 0.05). The highest frequency of Cu-tolerant bacteria was found in the Cu-polluted soil of South Chagres, which is the soil with the highest Cu content, while the lowest rate was found in the non-polluted soil from La Vinilla. These results revealed that Cu-tolerant cultivable bacteria in Cu-polluted soils were approximately 13 to 46 fold higher than in the non-polluted soil (Table 1).

**Table 1:** Number of heterotrophic and copper-tolerant cultivable bacteria of the agricultural soils

**Site**	**Log CFU g**^**-1**^**dry weight soil**^**a**^		**Cu-tolerant/total CFU**
	**Total**	**Cu-tolerant**	**(%)**
North Chagres	7.34 (0.04)	5.87 (0.04)	3
South Chagres	7.07 (0.05)	6.43 (0.15)	23
Ñilhue	7.23 (0.01)	6.34 (0.20)	14
La Vinilla	7.14 (0.03)	4.77 (0.05)	0.4

^a^ Standard deviations are indicated in parentheses.

### Characterization of Cu-resistant bacterial isolates

Cu-resistant bacteria were isolated from the three Cu-polluted soils from the Aconcagua valley. A representative collection of 92 bacterial strains (29 to 31 from each Cu-polluted soil) were isolated by enrichment in R2A medium containing Cu^2+^ (0.8 mM). The soil bacteria isolated were challenged with successive Cu^2+^ concentrations from 0.8 to 4.7 mM in LPTMS medium. A marked decrease in the cells number was observed in the medium containing Cu^2+^ (2.8 mM). Eleven bacteria that were capable of growing in the presence of Cu^2+^ (2.8 mM) were selected from the 92 isolates for further studies. Two bacterial strains isolated from Ñilhue were capable of tolerate 3.5 mM of Cu^2+^. Three isolates from South Chagres tolerate 3.5 mM of Cu^2+^. Six strains from North Chagres were capable of tolerate 2.8 mM of Cu^2+^. The Cu-resistant isolates were challenged to heavy metals for the determination of the MIC values. Five of the eleven Cu-resistant strains isolated (strain C21 from North Chagres, strains A32 and A55 from South Chagres; strains O4 and O12 from Ñilhue) showed also tolerance to Co^2+^, Ni^2+^, Zn^2+^, Hg^2+^ and CrO_4_^2-^ (Table 2). These five broad-range heavy metal resistant bacteria should possess diverse mechanisms for heavy metal resistance. Therefore, these isolates were selected for further characterization. Strains that were capable to grow in presence of 0.5 mM of Cu^2+^, Co^2+^, Ni^2+^, Zn^2+^ or CrO_4_^2-^ and 0.05 mM of Hg^2+^ were recorded as tolerant. Strain O12 showed a high MIC to Cu^2+^ (4.7 mM), Co^2+^ (2.5 mM), Ni^2+^ (17 mM), Zn^2+^ (8.5 mM) and Hg^2+^ (0.4 mM). Strain A32 and A55 showed a high MIC to Cu^2+^ (3.9 mM), Co^2+^ (2.5 mM), Ni^2+^ (17 mM), Zn^2+^ (8.5 mM) and Hg^2+^ (0.4 mM). Strain O4 showed a high MIC to Cu^2+^ (3.9 mM), CrO_4_^2-^ (4.3 mM), Co^2+^ (2.5 mM), and Ni^2+^ (8.5 mM). Strain C21 showed a high MIC to Cu^2+^ (3.1 mM), CrO_4_^2-^ (4.3 mM) and Co^2+^ (0.8 mM). All the strains had a low MIC to Cd^2+^ (lower than 0.4 mM), indicating that these strains were not resistant to this heavy metal.

**Table 2:** Minimum inhibitory concentration of heavy metal for soil bacterial isolates

**Strain**	**MIC (mM)**						
	**Cu**^**2+**^	**Co**^**2+**^	**Ni**^**2+**^	**Zn**^**2+**^	**Cd**^**2+**^	**Hg**^**2+**^	**CrO**_**4**_^**2-**^
O12	4.7	2.5	17	8.5	<0.4	0.4	<0.4
A32	3.9	2.5	17	8.5	<0.4	0.4	<0.4
A55	3.9	2.5	17	8.5	<0.4	0.4	<0.4
C21	3.1	0.8	0.9	<0.8	<0.4	0.1	4.3
O4	3.9	2.5	8.5	<0.8	<0.4	0.1	4.3
*C. metallidurans* MSR33^a^	3.8	20	6	17	2.5	0.1	0.7

^a^ Rojas *et al*. [31].

### Identification of Cu-resistant isolates

**Figure 3:**
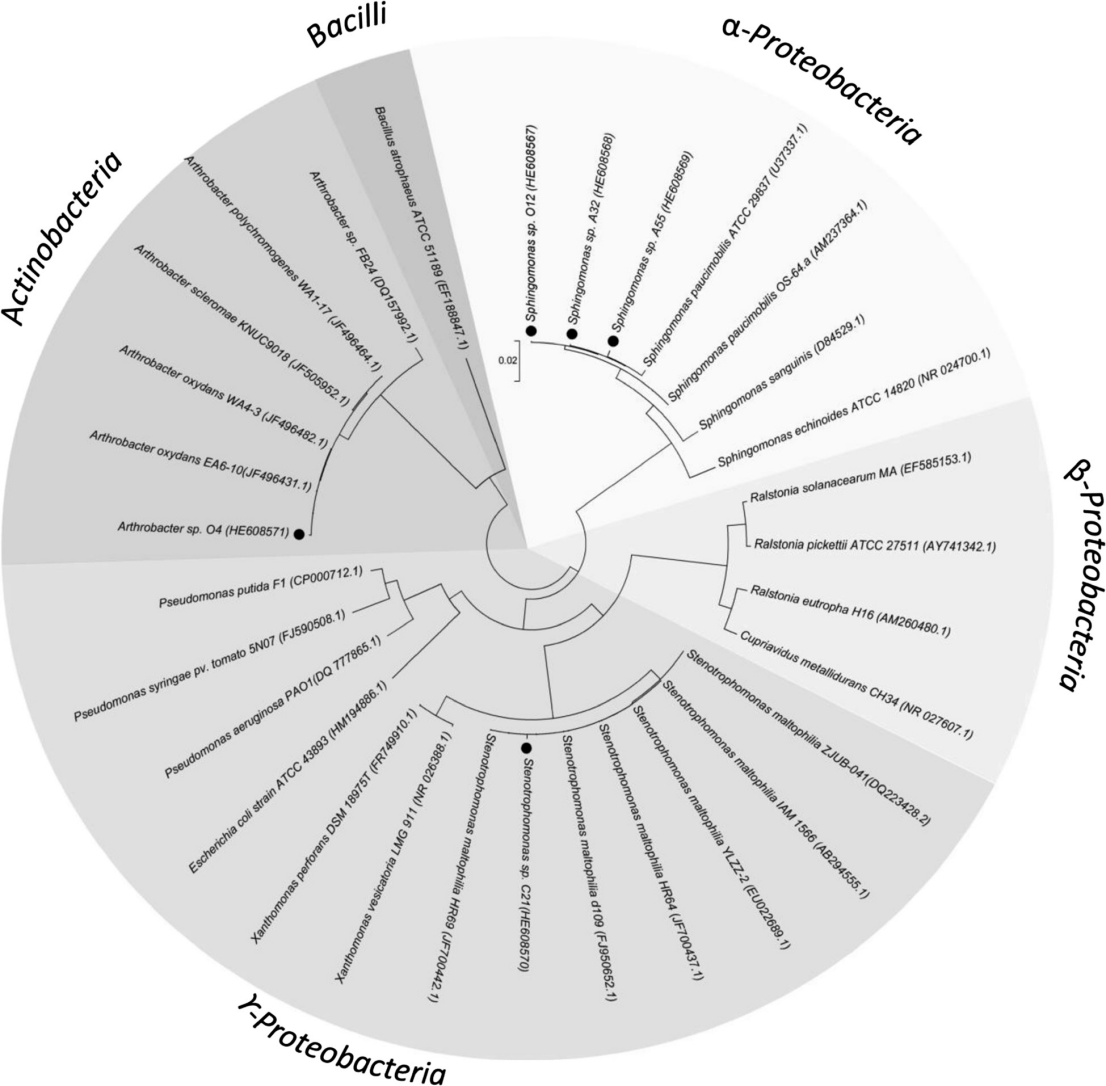
**Identification of bacterial isolates by 16S rRNA gene sequence analysis.** The phylogenetic tree was constructed using neighbor-joining method. Values of 1000 bootstrap replicates above 60% are given at the branching point. Sequences of the bacterial isolates *Sphingomonas* sp. strain O12, *Sphingomonas* sp. strain A32, *Sphingomonas* sp. strain A55, *Stenotrophomonas* sp. strain C21 and *Arthrobacter* sp. strain O4 are highlighted (black circles). Vertical bar represents 0.02 units of evolutionary distance.

For bacterial identification, comparative 16S rRNA gene sequence analyses of the bacterial isolates were used. The results indicated that isolates O12, A32 and A55 belong to the *Sphingomonas* genus*,* showing a high 16S rRNA gene sequence similarity (98%) to *Sphingomonas paucimobilis.* Isolate C21 was identified as a *Stenotrophomonas* strain, showing a high 16S rRNA gene sequence similarity (98%) to *Stenotrophomonas maltophilia*. Isolate O4 was identified as an *Arthrobacter* strain, with high 16S rRNA gene sequence similarity (99%) to *Arthrobacter oxydans*. The 16S rRNA gene sequences of the isolates and other bacteria including strains from *Stenotrophomonas*, *Sphingomonas* and *Arthrobacter* genera were used to build a phylogenetic tree (Figure 3). Strains O12, A32 and A55 are closely related to *Sphingomonas paucimobilis* strain OS-64.a. Strain C21 is closely related with *Stenotrophomonas maltophilia* strains HR69 and d109. Strain O4 is closely related with the Gram-positive bacteria *Arthrobacter oxydans* WA4-3 and *Arthrobacter oxydans* EA6-10 (Figure 3).

### PCR detection of heavy metal determinants in genomic DNA from bacterial isolates

**Figure 4:**
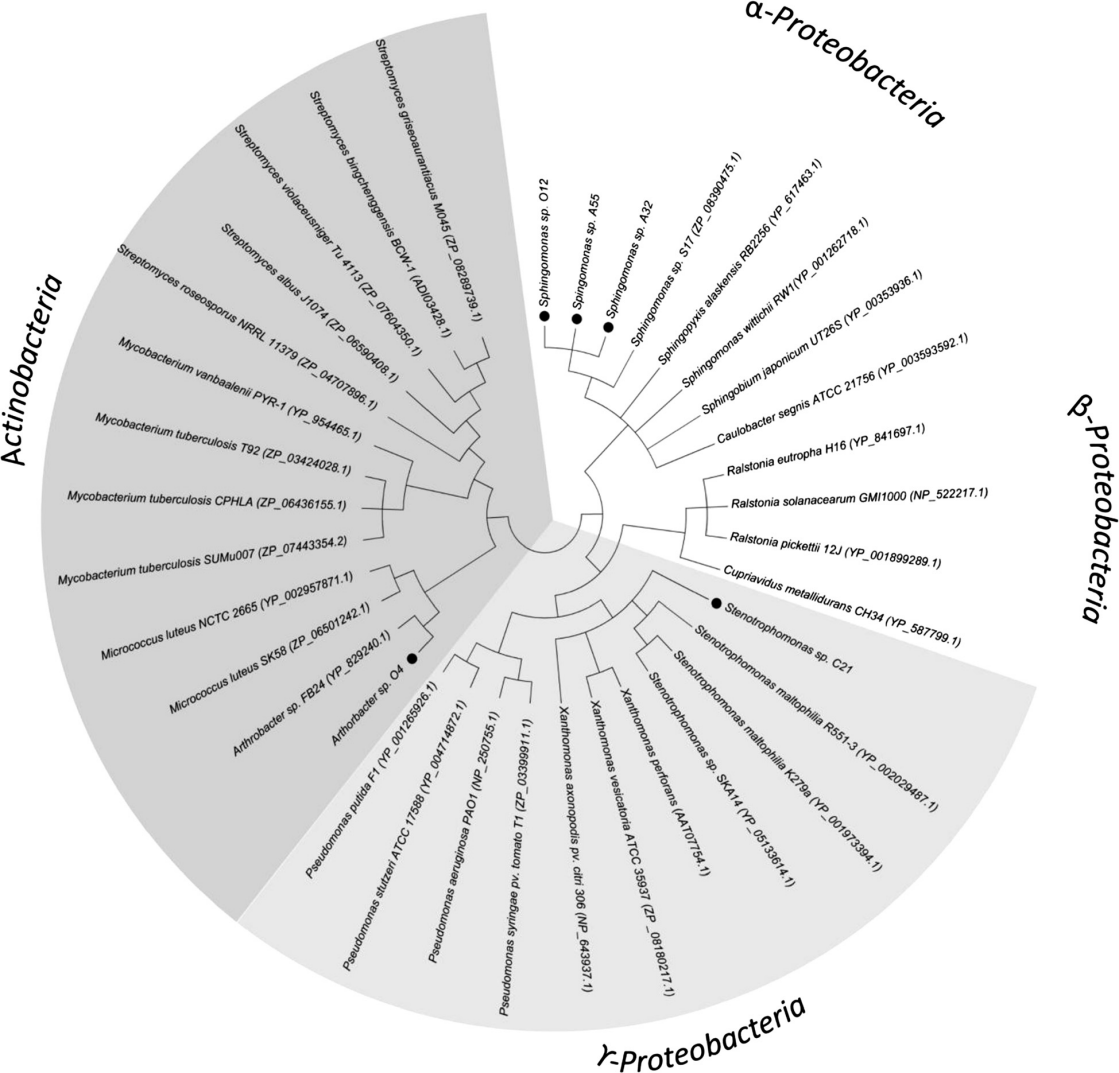
**Phylogenetic tree showing the relatedness of multi-copper oxidase CopA of the bacterial isolates.** The phylogenetic tree was constructed using neighbor-joining method. Values of 1000 bootstrap replicates above 50% are given at the branching point. Sequences of CopA proteins of the bacterial isolates *Sphingomonas* sp. strain O12, *Sphingomonas* sp. strain A32, *Sphingomonas* sp. strain A55, *Stenotrophomonas* sp. strain C21 and *Arthrobacter* sp. strain O4 are highlighted (black circles).

The presence of the *copA* gene encoding a multi-copper oxidase in the bacterial isolates was studied by PCR using the Coprun primers. The bacterial strains O12, A32, A55, C21 and O4 possess the *copA* genes. The PCR products varied between 1000–1200 bp. The CopA protein sequences were aligned with CopA sequences belonging to Cu-resistant bacteria and were used to construct a phylogenetic tree (Figure 4). Sequence analyses indicate that the *copA* genes of the isolates encode multi-copper oxidases that are involved in Cu resistance but that are not associated to degradation of phenolic compounds or polymers. The CopA protein of *Sphingomonas* sp*.* strains O12, A32 and A55 are closely related to CopA of other *α-Proteobacteria*, sharing high similarity (93%) with CopA from *Sphingomonas* sp. S17. The CopA from *Stenotrophomonas* sp. strain C21 belongs to the *Stenotrophomonas* and *Xanthomonas* CopA branch of the γ-*Proteobacteria* and is closely related to CopA from *Stenotrophomonas maltophilia* R551-3 (67% similarity). The CopA of strain *Arthrobacter* sp. O4 is closely related to the CopA of *Actinobacteria* and possess a 68% similarity with CopA from *Arthrobacter* sp. strain FB24.

Other heavy metal determinants were studied by PCR using specific primers for *merA* (Hg^2+^ resistance), *merB* (organomercurial resistance) and *chrB* (CrO_4_^2-^ resistance) genes based on *C. metallidurans* CH34 sequences. Using these specific primers, the *merA*, *merB* and *chrB* genes were not detected in the five Cu-resistant bacterial strains.

### Detection of plasmids in bacterial isolates

**Figure 5:**
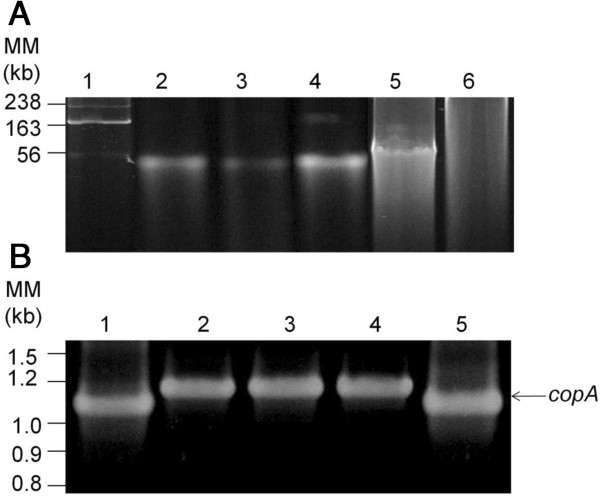
**Detection of plasmids encoding *copA *genes in copper-resistant bacterial isolates.A**. Agarose gel electrophoresis of plasmids isolated from *Sphingomonas* sp. strain O12 (lane 2) *Sphingomonas* sp. strain A32 (lane 3), *Sphingomonas* sp. strain A55 (lane 4) and *Stenotrophomonas* sp. strain C21 (lane 5). No plasmid was observed in *Arthrobacter* sp. strain O4 (lane 6). *C. metallidurans* strain MSR33 that carries the pMOL30, pMOL28 and pTP6 plasmids was used as a positive control (lane 1). **B**. Detection of *copA* gene in plasmids of bacterial isolates. The *copA* gene was detected in *Sphingomonas* sp. strain O12 (lane 2), *Sphingomonas* sp. strain A32 (lane 3), *Sphingomonas* sp. strain A55 (lane 4) and *Stenotrophomonas* sp. strain C21 (lane 5). *C. metallidurans* strain MSR33 (lanes1) was used as a positive control.

*Sphingomonas* sp. strain O12, *Sphingomonas* sp. strain A32, *Sphingomonas* sp. strain A55 and *Stenotrophomonas* sp*.* strain C21 possessed plasmids (Figure 5). Plasmids were no detected in *Arthrobacter* sp. strain O4. The plasmids of these four bacterial isolates contained the *copA* gene encoding a multi-copper oxidase (Figure 5).

## Discussion

In this report, the bacterial communities in long-term Cu-polluted agricultural soils from Aconcagua valley, central Chile, were studied and compared with the bacterial community of a non-polluted agricultural soil. The bacterial DGGE profiles showed high similarity (approximately 80%) among Cu-contaminated soils suggesting a low variation in the dominant bacterial groups in these soils. A similar number of bands and banding pattern was observed by DGGE fingerprints in polluted and non-polluted soils. Despite of the difference in the Cu content in soils, DGGE studies presented a similar Shannon diversity index (*H’*) and richness suggesting that the presence of high copper concentration and differences in other soil properties did not affect the diversity of the dominant groups of the bacterial communities detected by DGGE. These results are in agreement with a previous report of soils from abandoned Cu mines from South Australia that show a low impact of Cu on the dominant microbial diversity [35]. Probably, bacterial communities from long-term Cu-polluted soils are well adapted to the high Cu content. Short-term Cu pollution in soil induces significant modifications in bacterial community structure, but these changes were resilient after a few weeks or months [9]. Our results are in agreement with previous studies showing that Cu, Pb and Zn did not change significantly the bacterial diversity after long-term contamination [36,37].

The *copA* gene that encodes for the multi-copper oxidase is one of the main genetic determinants involved in Cu-resistance [1,25,26]. In this report, the presence of *copA* gene was studied in metagenomic DNA from agricultural soils. The c*opA* gene was detected in the three Cu-polluted soils. In contrast, the *copA* gene was not detected in metagenomic DNA from soils with low Cu content. The number of heterotrophic cultivable bacteria was constant in all agricultural soils, whereas, the number of Cu-resistant heterotrophic bacteria was significantly higher in Cu-polluted soils than in the non-polluted soil. These results suggest that the presence of high levels of Cu in Aconcagua valley soils is exerting a selective pressure on the bacterial communities, which favors the selection of Cu-tolerant bacteria.

Cu-tolerant bacteria were isolated from the Cu-polluted agricultural soils. Most of bacterial isolates were not capable to grow in LPTMS medium supplemented with Cu^2+^ (2 mM). Our results are consistent with a previous study that indicates that the number of resistant bacteria decreased at higher Cu^2+^ concentration [38]. Five isolates, *Sphingomonas* sp. strain O12, *Sphingomonas* sp. strain A32, *Sphingomonas* sp. strain A55, *Stenotrophomonas* sp. strain C21 and *Arthrobacter* sp. strain O4, were resistant to high Cu^2+^ concentration (≥ 2.8 mM) and other heavy metals. Bacteria that tolerate Cu concentrations higher than 2 mM should possess an effective resistance system for Cu detoxification. The isolates *Sphingomonas* sp. strain O12, *Sphingomonas* sp. strain A32 and *Sphingomonas* sp. strain A55, showed a high MIC to Cu^2+^ (ranged from 3.9 to 4.7 mM), Co^2+^ (2.5 mM), Ni^2+^ (17 mM), Zn^2+^ (8.5 mM) and Hg^2+^ (0.4 mM) (Table 2). The MIC of the heavy metal resistant bacterium *C. metallidurans* strain MSR33 to Cu^2+^, Co^2+^, Ni^2+^, Zn^2+^ and Hg^2+^ is 3.8, 20, 6.0, 17 and 0.1 mM, respectively. *Stenotrophomonas* sp. strain C21 and *Arthrobacter* sp. strain O4 showed a high MIC to Cu^2+^ (ranged from 3.1 to 3.9 mM) and CrO_4_^2-^ (4.3 mM). *C. metallidurans* strain MSR33 has a MIC to CrO_4_^2-^ of 0.7 mM. These high levels of heavy metal resistances may be useful for surviving and adapting to acute heavy metal contamination events in the soil. The mechanisms involved in heavy metal resistance of the strains isolated should be studied. *Sphingomonas macrogoltabidus* strain S1n isolated from rhizosphere of *Alyssum murale,* showed a high MIC to Cu^2+^ (5 mM) and Ni^2+^ (15 mM) [38]. *Stenotrophomonas maltophilia* strain SM777 isolated from a contaminated culture, showed high MIC to Cu^2+^ (5 mM), Ni^2+^ (10 mM), Zn^2+^ (4 mM), and Hg^2+^ (0.05 mM) [39]. *Arthrobacter* sp. strain E9 isolated from a battery-manufacturing contaminated site, showed a high MIC to Cu^2+^ (5.8 mM), Co^2+^ (2.5 mM), Zn^2+^ (3 mM) and Hg^2+^ (0.06 mM) [40]. *Arthrobacter* sp. strain S189 isolated from rhizosphere of *Alyssum murale,* showed a high MIC to Cu^2+^ (10 mM), Co^2+^ (5 mM), Ni^2+^ (15 mM), Zn^2+^ (10 mM) and Hg^2+^ (0.5 mM) [38].

The high level of heavy metal resistance of *Sphingomonas* sp. strain O12, *Sphingomonas* sp. strain A32, *Sphingomonas* sp. strain A55, *Stenotrophomonas* sp. strain C21 and *Arthrobacter* sp. strain O4 suggests that these strains possess diverse heavy metal determinants. The use of specific primers based on heavy metal determinants from *C. metallidurans* strain MSR33 did not yield amplicons. In future studies, the presence of heavy metal resistance genes will be studied using more general primers.

In this study, the presence of multi-copper oxidase gene was evaluated in the five isolates using degenerated primers designed for *copA* sequences from *Proteobacteria*. The presence of multi-copper oxidase *copA* genes in both Gram-negative and Gram-positive bacteria was determined. The presence of *copA* gene in both bacterial groups suggests that the *cop* system involved in Cu-resistance could be widespread in soil probably through horizontal genes transfer among soil bacteria. The sequence analysis indicates that the CopA proteins from *Sphingomonas* isolates (O12, A32 and A55), *Stenotrophomonas* strain (C21) and *Arthrobacter* strain (O4) are closely related to CopA from *Sphingomonas*, *Stenotrophomonas* and *Arthrobacter* strains, respectively. The *copA* genes of the fives isolates encode multi-copper oxidases that oxidize Cu(I) to Cu(II) but not phenolic compounds or polymers as other multi-copper oxidases reported [41,42].

Phylogenetic analyses of 16S rRNA gene sequences indicate that the isolates belong to *Sphingomonas*, *Stenotrophomonas* and *Arthrobacter* genera. The phylogenetic tree obtained from the sequence analysis of 16S rRNA gene was similar to those results predicted from the sequence analysis of CopA protein (Figure 3 and 4), showing a high concordance between structural and functional genes.

Mobile genetic elements (MGE) could be involved in the spreading of Cu resistance determinants, facilitating the adaptation of bacterial communities to copper [43]. Bacteria exposed to copper for a long period of time may acquire MGE such as plasmids carrying copper determinants and, therefore, they become copper-resistant bacteria [43-45]. In agreement with this hypothesis, this study showed the presence of the *copA* gene in metagenomic DNA from the three Cu-polluted soils and the absence of *copA* gene in metagenomic DNA from the non-polluted soil. This study demonstrates that Gram-negative Cu-resistant strains isolated from long-term Cu-contaminated soils carried plasmid with Cu-resistance determinants. The presence of plasmids encoding *copA* genes in *Sphingomonas* sp. strain O12, *Sphingomonas* sp. strain A32, *Sphingomonas* sp. strain A55 and *Stenotrophomonas* sp. C21 (Figure 5) confirm that MGE are involved in copper resistance in these isolates. The *copA* (*pcoA)* genes encoding multi-copper oxidases have been characterized in plasmids such as pPT23D, pRJ1004 and pMOL30 from *Escherichia coli* RJ92, *Pseudomonas syringae* pv. *tomato* PT23 and *Cupriavidus metallidurans* CH34, respectively [20,21,24]. The multi-copper oxidase *copA* gene was present in the genome of the Gram-positive bacterium *Arthrobacter* sp. O4, but plasmids were not detected in this strain. The CopA protein sequence from *Arthrobacte*r sp. O4 possesses a high similarity (68%) with the multi-copper oxidase gene of *Arthrobacter* sp. FB24, which is located in a plasmid [46,47]. As plasmid isolation in some bacterial strains is difficult, the presence of the *copA* gene from *Arthrobacte*r sp. O4 in a plasmid could not be excluded.

## Conclusions

This study have shown that the bacterial community diversity of agricultural soil of central Chile analyzed by DGGE was similar in Cu-polluted and non-polluted soils. The *copA* gene encoding multi-copper oxidase was detected only in metagenomic DNA of Cu-polluted soils suggesting that *copA* genes are widely spread in contaminated environments. Cu-resistant bacteria were isolated from these long-term polluted soils. The MIC studies on bacterial isolates indicated that Cu-resistant bacteria were also resistant to other heavy metal such as Ni^2+^, Hg^2+^ and CrO_4_^2-^. The *copA* gene was detected in the Cu-resistant *Sphingomonas*, *Stenotrophomonas* and *Arthrobacter* strains isolated from the polluted agricultural soils. The multi-copper oxidase *copA* gene is located in plasmids in the four *Sphingomonas* and *Stenotrophomonas* strains suggesting that MGEs may spread Cu-resistance determinants in these soils. This study contributes to the understanding of the effect of long-term Cu-pollution on the bacterial community of agricultural soils and to the characterization of novel Cu-resistant bacterial isolates from agricultural soils from the Aconcagua valley.

## Competing interests

The authors have declared that no competing interests exist.

## Authors' contributions

Conceived and designed the experiments: FA, CY, MG, MS. Soil sampling: FA, CY, GB. Performed the experiments: FA, MG, LAR, GB. Analyzed the data: FA, CY, GB, MG, LAR, MS. Contributed reagents/materials/analysis tools: MS, MG, CY. Wrote the paper: FA, LAR, MS. All authors read and approved the final manuscript.
